# A Retrospective Analysis Evaluating the Outcome of Parenteral Nutrition in the Treatment of Anorexia Nervosa in Korea

**DOI:** 10.3390/jcm9113711

**Published:** 2020-11-19

**Authors:** Jeong-Kyung Ko, You-Kyung Lee, Jong Chun Na, Dong-Yeon Kim, Youl-Ri Kim

**Affiliations:** 1 Institute of Eating Disorders and Mental Health, Inje University, Seoul 14551, Korea; jen52787@gmail.com (J.-K.K.); leeyoukyung260@gmail.com (Y.-K.L.); 2 Department of Internal Medicine, Division of Cardiology, Seoul Paik Hospital, Inje University, Seoul 14551, Korea; drnah1970@hanmail.net (J.C.N.); dr.dongyeonkim@gmail.com (D.-Y.K.); 3 Department of Neuropsychiatry, Seoul Paik Hospital, Inje University, Seoul 14551, Korea

**Keywords:** anorexia nervosa, treatment, parenteral nutrition, inpatient, eating disorders

## Abstract

The objective of this study was to investigate the clinical efficacy of parenteral nutrition (PN) as supplemental feeding for patients with anorexia nervosa (AN). This study was conducted by reviewing the medical records of patients with AN who were hospitalized at a non-specialized ward. A total of 129 patients with AN were recruited, consisting of 67 patients received PN with oral refeeding and 62 patients received oral refeeding alone. We compared the weight gain at discharge and after discharge between the groups. As a result, at admission, the patients given supplementary PN had lower body mass indices and lower caloric intake than the patients without PN. The mean duration of PN was 8.5 days, which amounted to about a third of the average hospital stay with no difference between the groups. Both groups had similar weight gains during hospitalization, but the patients with PN had higher weight gains than the patients without PN at one and three months after discharge. In conclusion, the results suggest that supplementary PN in the early stage of refeeding might initiate weight gain in AN when nasogastric tube feeding is not possible. Randomized controlled trials are needed to be further tested of PN in treatment of AN.

## 1. Introduction

Anorexia nervosa (AN) is a highly distinctive serious mental disorder with low body weight due to severe dietary restriction or other weight-loss behaviors aimed at preventing weight gain (e.g., purging and excessive physical activity) [1]. The importance of early weight gain for long-term recovery has been demonstrated in inpatient settings [2,3,4].

Though standard treatment guidelines recommend caring for all people with AN in contact with specialist services [5,6,7], there are no specialized eating disorder wards in a general hospital and only a few trained clinical staff in the field of eating disorders in Korea. Therefore, patients with AN in need of acute hospitalization are commonly managed within a closed psychiatric ward in Korea. The patients with AN are treated together with individuals with other psychiatric disorders and this adds a level of challenge to their overall clinical management. Few specialized resources for AN but relatively adequate numbers of hospital beds in Korea [8] prompted us to treat patients with AN in an open psychiatric ward. In this environment, we developed supplemental parenteral feeding by necessity, not by design.

Parenteral nutrition (PN) and nasogastric tube (NG) feeding have been considered alternatives for oral feeding in patients with severe and medically complicated AN [9]. Indeed, NG feeding should represent the primary route of nutritional rehabilitation as an alternative method in addition to oral refeeding [10]. NG feeding is relatively safe, but many patients complain of abdominal pain and discomfort, and some patients remove the nasogastric tube by themselves, which was difficult to manage in a non-specialized ward. A feasible alternative to NG is PN for severely malnourished patients with AN in a non-specialized ward, though the disadvantages are its invasiveness and more side effects than with NG feeding [11,12,13]. Studies in Italy and Japan reported PN as an optional treatment to provide higher calories and more rapid advancement in caloric intake in patients who refuse oral refeeding [11,14], but there has been controversy regarding the potential risk of total parenteral nutrition (TPN) delivered through a central vein catheter [15,16]. For this reason, the use of TPN has not been generally accepted as a standard nutritional approach in eating disorder units [17]. Peripheral PN delivered by an intravenous catheter in the forearm ensures essential caloric intake in a relatively safe way compared to TPN. It might also have the benefit to overcome resistance to refeeding in the early stage of treatment as gastrointestinal symptoms are common and bothersome in AN [18].

The aim of this study was to examine the efficacy and safety of supplementary PN refeeding in patients with AN in a non-specialized setting. We used weight differences, the weight gain rate, body mass index (BMI), and medical parameters as outcome determinants. We hypothesized that supplemental PN refeeding would affect short-term weight gain, which initiate long-term weight restoration.

## 2. Methods

### 2.1. Participants

**Figure 1:**
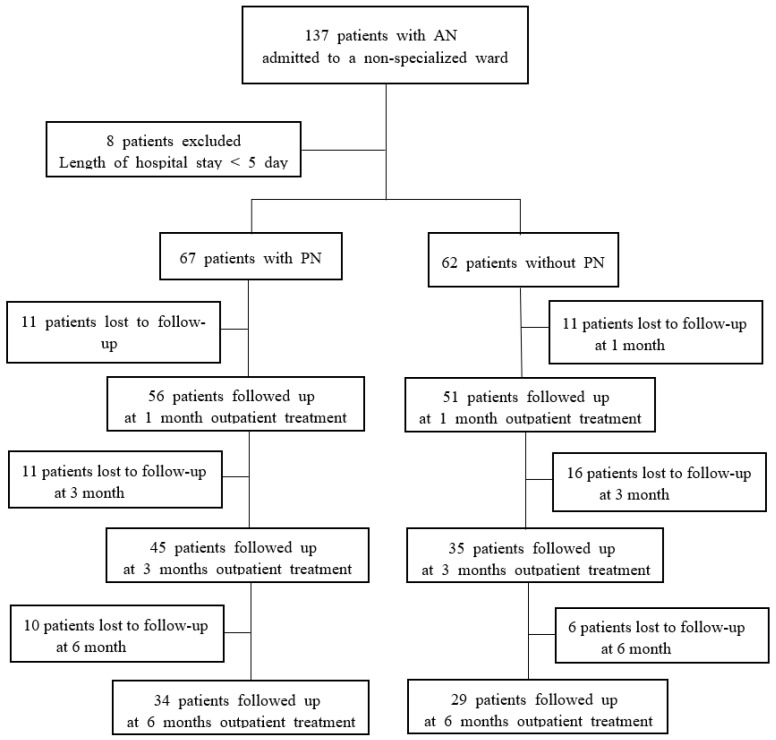
Flow-diagram of the study participants. Abbreviations: AN, anorexia nervosa; PN, parenteral nutrition.

In this study, we retrospectively collected data by reviewing medical records. All of the patients with AN who were consecutively admitted to a non-specialized psychiatric open ward between March 2010 and March 2016 in a university hospital, Seoul, South Korea, were included in the study. Patients hospitalized for fewer than five days were excluded from the analyses (*n* = 8). Diagnoses were made according to the Diagnostic and Statistical Manual of Mental Disorders, Fifth Edition (DSM-5) [19]. The diagnosis of AN in 129 patients involved restricted (RAN, *n* = 82) or binge-eating/purging (BPAN, *n* = 47) types by the DSM-5 (Figure 1). All participants were Korean, 95.3% (*n* = 123) were female, and 32.6% (*n* = 42) were below the age of 16. The characteristics of the participants are shown in Table 1.

The retrospective study was approved by the Ethical Committee in the university (2015359-02).

### 2.2. Treatment Protocol for Eating Disorders in the Non-Specialized Ward

The setting in the psychiatric open ward included supervised meals and snacks implemented by a team of psychiatrists, nurses, and psychologists with supervising meals/snacks without complete monitoring after meals. Due to the shortage of psychotherapists to provide specialized programs for AN during admission, education and supportive psychotherapy were provided by trainee doctors and nurses in the ward. If the patient refused NG feeding, PN was suggested as an alternative. Patients showed less resistance to having a catheter inserted into their forearm vein than feeding by a nasogastric tube, and no patient chose NG feeding in the ward. PN was initiated if a patient refused to eat meals required for a basal metabolic rate. Along with oral intake, the total caloric intake started between 500 and 1000 kcal per day and was gradually increased, but was limited to under 1400 kcal per day. Peripheral PN was used for ≤ 2 weeks. In every case, PN was given through venous access in the forearm.

The PN solution (Winuf Peri Inj^®^, JW Pharmaceutical, Seoul, Korea) was a complex nutritional formula that contained nutrients for peripheral infusion. The ingredients per bag (1085 mL) were calcium chloride dihydrate 0.73 g, glucose monohydrate 143.02 g, glycine 10.3 g, L-alanine 20.7 g, L-arginine 11.5 g, L-histidine 4.8 g, L-isoleucine 6 g, L-leucine 7.3 g, L-lysine hydrochloride 7.25 g, L-methionine 4 g, L-phenylalanine 5.6 g, L-proline 6.8 g, L-serine 5 g, L-threonine 4.2 g, L-tryptophan 1.8 g, L-tyrosine 0.4 g, L-valine 5.8 g, magnesium sulfate heptahydrate 2.457 g, medium-chain triglyceride 50 g, potassium chloride 4.528 g, purified fish oil 40 g, purified olive oil 50 g, purified soybean oil 60 g, sodium acetate hydrate 5.548 g, sodium glycerophosphate 4.193 g, zinc sulfate heptahydrate 0.023 g.

During the hospital stay, the daily caloric supply of 1200 to 1400 total kcal/day at admission was increased 200 to 400 kcal/d every 3 to 4 days to 60 kcal/kg/day by discharge. All oral caloric intake consumed as food was recorded in the nursing records. As brief inpatient care followed by intensive outpatient care could achieve similar efficacy to that of longer hospital stays [20], hospital discharge was ordered when a patient was medically stable and agreed to continue treatment on an outpatient basis.

### 2.3. Outpatient Treatment

Outpatient treatment was conducted in the eating disorder outpatient clinic in the hospital. Caloric intake was advanced further with therapists after discharge. The target weight for outpatient treatment was age-adjusted BMI for adolescents or the World Health Organization standard BMI of 18.5 kg/m^2^ or higher in adults.

The outpatient treatment was not different whether the patient took PN or not. Treatment modalities other than nutritional therapy included standard psychotherapies for AN such as individual cognitive behavioral therapy, the Maudsley Model of anorexia nervosa [21], and specialist supportive clinical management [22] for adults and family therapy for adolescents. Psychotropic medications were prescribed for the patients, if necessary. Antidepressants for depression or obsessive-compulsive symptoms, or antipsychotics for severe anxiety or delusional fear of food or weight gain were initiated during the hospital stay after the patients were medically stabilized.

### 2.4. Clinical Data Collection

The clinical data collected from reviewing the medical records included the length of hospital stay; the weight and height at admission, discharge, and 1, 3, and 6 months after discharge; and the calories supplied during hospitalization. The primary outcome measure was BMI and weight restoration at discharge, and 1, 3, and 6 months after discharge. The weight gain rate was also calculated (kg/week). The denominators differed depending on the period resulting from missed appointments or patients lost to follow-up.

Laboratory testing was performed at admission and discharge, and additionally if needed. Psychological assessments were obtained at admission, including the eating disorder examination questionnaire (EDE-Q) [23], the state-trait anxiety inventory (STAI) [24], and the Beck depression inventory (BDI) [25].

### 2.5. Statistical Analyses

We examined the differences in weight gain between those who took supplemental PN (67 patients with PN) and those who received oral intake only (62 patients without PN) during hospitalization. To compare nutritional therapy between the groups, independent t-tests were applied for the continuous variables and the Chi-squared test was used for the categorical variables. The effects of PN on the medical findings at discharge were examined with the repeated-measures analysis of variance (ANOVA) model according to the group (patients with PN and patients without PN) and period (admission and discharge) including initial BMI as a covariate. Paired t-tests were used for the group-specific comparisons of medical findings between the periods. For comparisons of the BMI and rate of weight gain between the groups, ANOVA with a block design was applied to control for the effect of subtypes as the restriction type was higher in the PN group. The effect sizes were calculated with Cohen’s d [26] or partial eta-squared (Δη^2^), if appropriate. Δη^2^ was interpreted as described as small (<0.01), medium (<0.059), or large (<0.138) [26].

All data were analyzed with SPSS 23.0 statistics software (IBM SPSS Statistics for Windows, Version 23.0. IBM Corp, Armonk, NY, USA). The level of significance was set at *p* < 0.05.

## 3. Results

### 3.1. Clinical Characteristics of Patients Who Received Supplemental Parenteral Refeeding and Patients with Oral Refeeding Alone

The clinical features and severity of the participants upon admission are presented in Table 1. The patients with PN had lower current BMIs (t(127) = −6.21, *p* < 0.001, d = 1.10), the lowest ever BMIs (t(127) = −3.71, *p* < 0.001, d = 0.66), the highest ever BMIs (t(127) = −4.36, *p* < 0.001, d = 0.99), and also had fewer previous admissions (t(68) = −2.20, *p* = 0.031, d = 0.40), whereas the patients without PN were diagnosed with more binge-eating and purging type AN (χ^2^(1) = 24.94, *p* < 0.001). There were no significant differences in the EDE-Q subscales, depression and anxiety (*p* > 0.1). Among EDE-Q total, only eating concern was different between the groups (t(102) = −2.02, *p* = 0.046, d = 0.40). There was no difference in use of antidepressants between the PN (*n*= 49, 73.1%) and the non-PN groups (*n* = 47, 75.8%) (χ^2^(1) = 0.121, *p* = 0.840).

### 3.2. Nutritional Therapy in Patients Who Received Supplemental Parenteral Refeeding and Patients with Oral Refeeding Alone

The nutritional therapy provided during hospitalization is shown in Table 2. At admission, the oral caloric intake was higher in the patients without PN (1967.90 ± 483.26 kcal/day) than in the patients with PN (1327.61 ± 554.47 kcal/day, t(127) = −6.97, *p* < 0.001, d = 1.23), whereas there was a difference in calories in the meals (t(120) = −6.45, *p* < 0.001, d = 1.12) and snacks (t(127) = −3.77, *p* < 0.001, d = 0.66). Then, the patients with AN received supplemental PN (619.60 ± 265.51 kcal/day) for 8.49 ± 5.62 days. The mean duration of the hospital stay was 24.66 ± 11.61 days for the patients with PN and 21.65 ± 10.67 days for the patients without PN, with no difference between the groups (*p* > 0.1).

At discharge, the oral calories consumed were higher in the patients without PN (2400.00 ± 392.34 kcal/day) than in the patients with PN (2188.20 ± 643.96 kcal/day, t(110) = −2.27, *p* = 0.025, d = 0.39), whereas there was a difference in calories in the meals (t(99) = −2.67, *p* = 0.009, d = 0.46) but not in snacks (t(127) = −0.30, *p* = 0.766, d = 0.05).

There were no differences in patients who received psychotherapy during hospitalization [χ^2^(1) = 0.24, *p* = 0.621] between the groups.

### 3.3. Medical Findings at Admission and Discharge in Patients with Supplemental Parenteral Refeeding and Patients with Oral Refeeding Alone

The medical findings at admission and discharge in the patients with PN and the patients without PN are presented in Table 3. In the patients with PN, there were differences between the admission and discharge in white blood cell counts (t(56) = −2.94, *p* = 0.004), hemoglobin levels (t(56) = 3.42, *p* = 0.001), platelet counts (t(56) = −2.42, *p* = 0.019), serum sodium levels (t(41) = −2.81, *p* = 0.008), serum chloride levels (t(41) = −2.45, *p* = 0.019), and cholesterol levels (t(35) = 2.08, *p* = 0.045). In the patients without PN, there were differences between the admission and discharge in serum potassium levels (t(31) = −3.20, *p* = 0.003) and serum chloride levels (t(31) = −2.79, *p* = 0.009).

We examined the effects of the group (patients with PN and without PN), period (admission and discharge), and their interaction on the medical parameters using repeated-measures ANOVA after controlling for the level of BMI at admission. There were significant main effects of the group with regard to white blood cell counts (F (1, 94) = 4.90, *p* = 0.029), hemoglobin levels (F (1, 94) = 9.86, *p* = 0.002), serum potassium levels (F (1, 71) = 5.95, *p* = 0.017), serum chloride levels (F (1, 71) = 4.62, *p* = 0.035), and protein levels (F (1, 66) = 5.78, *p* = 0.019). There were significant main effects of the period with regard to hemoglobin levels (F (1, 94) = 4.25, *p* = 0.042), and platelet counts (F (1, 94) = 15.57, *p* <0.001). There were no significant interactions between the groups and periods in which the differences between the medical parameters at admission and discharge did not depend upon the group.

### 3.4. Safety of and Compliance with Parenteral Nutrition

No fatal complications related to PN were observed. Twelve patients in the PN group reported refeeding edema, which was the same number as in the group without PN (17.9% for patients with PN vs. 19.4% for patients without PN, (χ^2^(1) = 0.04, *p* = 0.833) (Table 2). No infections related to PN were reported. Two extremely underweight patients (BMI < 11 kg/m^2^) showed somatic delusions (e.g., “I felt a mass in my stomach”) with the fluid supplied by PN, which diminished with weight gain. The voluntary discharge time that patients wanted to be carried out did not differ between the groups (7.5% of the patients with PN vs. 16.1% of the patients without PN, χ^2^(1) = 2.35, *p* = 0.170) (Table 2).

### 3.5. Weight Gain in Patients Who Received Supplemental Parenteral Refeeding and Patients with Oral Refeeding Alone

**Figure 2:**
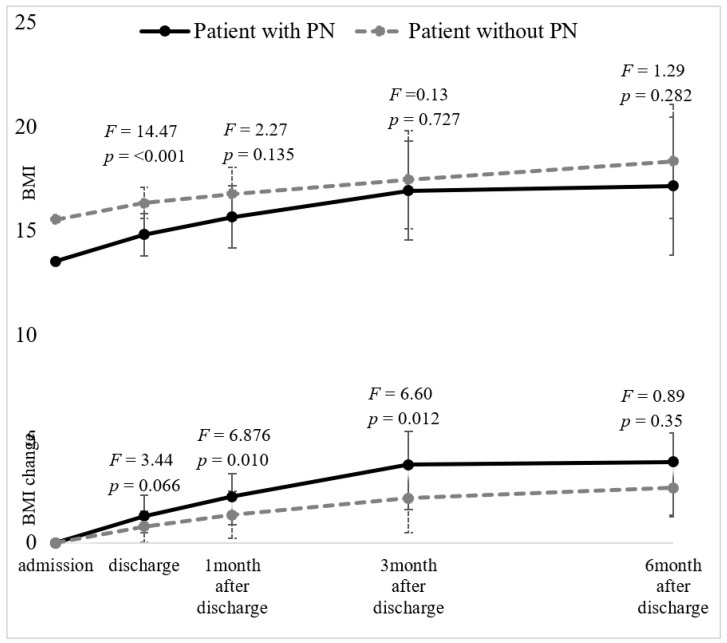
The comparisons of BMI and the BMI change controlled for the effect of AN subtypes between the patients with PN and the patients without PN at discharge and 1, 3 and 6 months after discharge. The bars indicate the mean ± standard error at each time point. Abbreviations: BMI, body mass index; PN, parenteral nutrition.

We analyzed the differences in weight gain and the rate of weight gain between the groups and the results are presented in Table 4 and Figure 2.

At discharge, though the patients with PN still had lower BMIs (14.79 ± 1.77 kg/m^2^) than the patients without PN with a large effect size (16.41 ± 1.68 kg/m^2^, F(127) = 14.47, *p* < 0.001, Δη^2^ = 0.096), their gained weight was 3.14 ± 2.57 kg compared to the patients without PN (2.19 ± 1.84 kg) during hospitalization, and the effect size of the group difference was small (F(127) = 3.44, *p* = 0.066, Δη^2^ = 0.025). There was no difference in the rate of gain between the groups (for patients with PN, 0.88 ± 0.90 kg/week vs. patients without PN, 0.79 ± 1.02 kg/week, *p* > 0.1) during hospitalization.

At one month after discharge, the BMI of the patients with PN was not different from that of the patients without PN (for patients with PN, 15.65 ± 1.99 kg/m^2^ vs. patients without PN, 16.81 ± 1.65 kg/m^2^, *p* > 0.1). The total weight gained was 5.43 ± 3.67 kg in the patients with PN and 3.65 ± 3.32 kg in the patients without PN, and the effect size of the difference was medium (F(105) = 6.95, *p* = 0.010, Δη^2^ = 0.060). There was no difference in the rate of gain between the groups (for patients with PN, 0.52 ± 0.70 kg/week vs. patients without PN, 0.33 ± 0.72 kg/week, *p* > 0.1).

At three months after discharge, the BMIs between the groups were not different (for patients with PN, 16.92 ± 2.54 kg/m^2^ vs. patients without PN, 17.58 ± 2.60 kg/m^2^, *p* > 0.1). The total weight gained was 9.25 ± 5.93 kg in the patients with PN and 5.89 ± 5.94 kg in the patients without PN, and the effect size of the difference was medium (F(78) = 6.33, *p* = 0.014, Δη^2^ = 0.074). The rate of weight gain was not different between the groups (for patients with PN, 0.42 ± 0.50 kg/week vs. patients without PN, 0.18 ± 0.54 kg/week, *p* > 0.1).

At six months after discharge, there were no differences in BMIs (for patients with PN, 17.14 ± 3.10 kg/m^2^ vs. patients without PN, 18.55 ± 2.79 kg/m^2^, *p* > 0.1). The total weight gained was 9.67 ± 8.50 kg in the patients with PN and 7.11 ± 6.65 kg in the patients without PN, which was not different between the groups (*p* > 0.1). There was no difference in the rate of gain between the groups (for patients with PN, 0.12 ± 0.33 kg/week vs. patients without PN, 0.14 ± 0.30 kg/week, *p* > 0.1).

The follow-up rate at three months was 68.2% in the PN group and 52.9% in the oral feeding group, and 50.7% and 48.3% at six months, respectively. There were no differences in the demographic characteristics or clinical severity between the followed patients and the patients lost due to attrition at six months (all *p*-values > 0.1). The only difference was found in the duration of hospitalization, in which the followed patients had longer hospital stays than the patients lost due to attrition (t (125) = −2.807, *p* = 0.006).

## 4. Discussion

This study retrospectively examined the clinical efficacy of PN refeeding on weight gain and laboratory findings in patients with AN. The patients given PN had lower BMI and caloric intake at baseline, but showed similar weight gains during hospitalization compared to the patients without PN.

We used peripheral PN for a relatively short period (8.5 ± 5.6 days) to decrease the risk of dislocation, infiltration, and infection. There was no report of any severe or dangerous complications related to PN, consistent with the study by Diamenti et al. [11]. Leg edema caused by fluid retention during the first phases of nutritional rehabilitation subsided with careful monitoring of the total fluid administration to the patients without PN. It has been reported that the clinical outcomes of long- and short-term PN administrations did not differ [11,14]. Furthermore, lack of use of the gut may promote bacterial translocation [27]. In a future study, the optimal length of PN administration needs to be determined.

Supplemental PN corrected laboratory value abnormalities, without a significant occurrence of refeeding syndrome. The serum hemoglobin levels were increased in PN group compared to the non-PN group at baseline, and rather normalized similar to the non-PN group at discharge. Patients without PN showed greater increases in serum potassium levels after refeeding, which seemed to be due to the higher proportion of binge eating and purging type in the group. Hypokalemia is a frequent manifestation of binge eating and purging AN [28].

In our data, the average caloric intake by discharge was <2500 kcal/d in both groups. The caloric prescription employed in the setting was relatively low considering the current recommendations [7]. In our setting, the calories were advanced further after discharge and averaged 0.88 kg/week for inpatients and 0.52 kg/week by one month, 0.42 kg/week by three months, and 0.12 kg/week by six months post-discharge in the PN group. Though these rates were similar to traditional treatment approaches where the mean weight gain in inpatient settings was 537 g/week in adults and 105 g/week in outpatient settings in adults [1,29]. The PN group had a tendency to gain more weight than the non-PN group with a small effect size. This was mainly due to a greater increase in calories during hospitalization in the PN group than in the non-PN group and also might have been partially caused by fluid retention as well as the sedentary activity level imposed by the intravenous hyperalimentation stand with a large, hanging drip-infusion bag in the PN group.

In our study, supplemental PN offers useful alternative for initiating treatment of AN to overcome initial resistance of refeeding and correct dangerous medical conditions, when NG feeding is not possible. The results of our study are consistent with the findings of Diamenti et al. [11], in which patients with supplemental PN had higher weight gains despite greater disease severity at baseline than patients without PN. Before applying PN intervention, we should consider the ethical aspects of artificial nutrition where nutrients and fluids be administered via the intravenous route, when an individual’s fluid and nutrient requirements cannot be covered via the enteral route [30].

The strengths of this study were the inclusion of all patients with AN consecutively admitted to a hospital with a follow-up six months after discharge. The study had several limitations that need to be acknowledged. Firstly, the study was not controlled. The features that contribute to provider decisions to use PN might contribute to some of the outcome measures. For example, the PN group’s lower weight, lowest ever weight, highest ever weight may contribute to the primary outcome measure of weight. However, in our experience, PN-treated patients were more malnourished at baseline and also showed higher rates of medical complications. Therefore, our results are meaningful in respect that PN induced weight gain for patients with greater severity of disease in the initial state of treatment comparable to less severe patients. Second, this study was conducted retrospectively, so the retrospective data retrieval was likely incomplete. Prospectively obtained data might be more accurate and reliable. Third, the duration of inpatient treatment was not enough. This early discharge was partially due to the Korean insurance system, where the coverage for hospitalization costs is the lowest among the Organization for Economic Co-operation and Development (OECD) countries. It resulted in premature discharge without reaching a target weight during hospitalization. Fourth, we did not separate the adolescents’ samples due to the small sample size. Fifth, the attrition rate was relatively high at three and six months. However, we believe that the patients maintained at six months do not represent a biased population as there was no difference in clinical characteristics and severity between the cases maintained and the cases lost to follow-up. Lastly, hospital lengths of stay varied though the follow-up time points, but which were structured as one, three, and six months following discharge.

In conclusion, the results suggest that supplementary PN in addition to oral refeeding may be an alternative way to gain weight effectively and initiate weight restoration in the early stage of treatment in a non-specialized ward, if NG feeding is not possible and PN is an ethically sound choice.

## Figures and Tables

**Table 1:** Clinical characteristics of AN patients with anorexia nervosa at admission.

	Patients with PN		Patients without PN		t or χ^2^	df	*p*	d
Variables	*n*	Mean (SD)	*n*	Mean (SD)				
Age at admission (years)	67	19.04 (7.94)	62	21.55 (6.64)	−1.93	127	0.055	0.34
BMI (kg/m^2^)	67	13.52 (1.93)	62	15.55 (1.75)	−6.21	127	<0.001 ***	1.1
Lowest ever BMI (kg/m^2^)	67	13.11 (1.82)	62	14.23 (1.58)	−3.71	127	<0.001 ***	0.66
Highest ever BMI (kg/m^2^)	67	19.06 (2.67)	62	22.24 (3.69)	−4.36	127	<0.001 ***	0.99
Duration of illness (months)	67	28.48 (42.69)	62	43.05 (41.94)	−1.95	127	0.053	0.34
Number of admissions	67	1.18 (.575)	62	1.84 (2.29)	−2.20	68	0.031 *	0.4
Binge-eating and purging type, *n* (%)	67	11 (16.4)	62	36 (58.1)	χ^2^ = 24.94	1	<0.001 ***	n/a
EDE-Q								
Total	56	10.39 (4.64)	48	11.28 (4.90)	−0.96	102	0.34	0.19
Restraint	56	3.32 (1.45)	48	2.89 (1.46)	1.51	102	0.135	0.3
Eating concern	56	2.23 (1.53)	48	2.87 (1.68)	−2.02	102	0.046 *	0.4
Weight concern	56	2.59 (1.53)	48	2.83 (1.50)	−0.80	102	0.425	0.16
Shape concern	56	2.2 3(1.41)	48	2.68 (1.57)	−1.55	102	0.124	0.3
Psychiatric comorbid symptoms								
BDI	60	24.50 (11.72)	58	27.81 (12.78)	−1.47	116	0.145	0.27
STAI-state	59	54.92 (13.04)	58	58.34 (10.98)	−1.53	115	0.128	0.28
STAI-trait	58	55.72 (13.47)	58	59.19 (11.33)	−1.50	114	0.137	0.28

Abbreviations: AN, anorexia nervosa; PN, parenteral nutrition; BMI, body mass index; EDE-Q, eating disorder examination questionnaire; BDI, Beck’s depression inventory; STAI, state-trait anxiety inventory; n/a, not applicable; d, Cohen’s d effect size. * *p* < 0.05, *** *p* < 0.001.

**Table 2:** Nutritional therapy for AN patients with and without PN during hospitalization.

Variables	Patients with PN (*n* = 67)	Patients without PN (*n* = 62)	t	df	*p*	d
At admission						
Oral feeding (kcal/d)	1327.61 (554.47)	1967.90 (483.26)	−6.97	127	<0.001 ***	1.23
Meal	1194.77 (517.72)	1706.45 (376.72)	−6.45	120	<0.001 ***	1.12
Snack	132.82 (188.60)	261.45 (198.84)	−3.77	127	<0.001 ***	0.66
PN (kcal/d)	619.60 (265.51)	n/a	n/a	n/a	n/a	n/a
Duration PN (days)	8.49 (5.62)	n/a	n/a	n/a	n/a	n/a
At discharge						
Total calories (kcal/d)	2188.20 (643.96)	2400.00 (392.34)	−2.27	110	0.025 *	0.39
Meal	1819.55 (511.95)	2008.06 (260.07)	−2.67	99	0.009 **	0.46
Snack	380.59 (244.01)	391.93 (179.06)	−0.30	127	0.766	0.05
Length of stay (days)	24.66 (11.61)	21.65 (10.67)	1.53	127	0.129	0.27
	*n* (%)	*n* (%)	χ^2^	df	*p*	
Refeeding edema	12 (17.9)	12 (19.4)	0.04	1	0.833	
Psychotherapy	64 (95.5)	58 (93.5)	0.24	1	0.621	
Voluntary discharge	5 (7.5)	10 (16.1)	2.35	1	0.170	

Note: Data are shown as mean (SD) if not otherwise specified. Abbreviations: AN, anorexia nervosa; PN, parenteral nutrition; n/a, not applicable; d, Cohen’s d, effect size. * *p* < 0.05, ** *p* < 0.01, *** *p* < 0.001.

**Table 3:** Medical parameters of AN patients with and without PN at admission and discharge.

	Group								Two-Way Repeated-Measures ANOVA						
	Patients with PN (*n* = 67)				Patients without PN (*n* = 62)				Main Effect					Interaction Effect	
Variables	Period		t(df)	*p*	Period		t(df)	*p*	Group			Period		F	*p*
	Admission	Discharge			Admission	Discharge			df	F	*p*	F	*p*		
Systolic BP (mmHg)	89.5 (13.0)	89.6 (8.6)	−0.05 (66)	0.963	93.4 (14.1)	93.9 (10.0)	−0.27 (60)	0.786	1.132	000	0.960	0.01	0.929	0.223	0.638
Diastolic BP (mmHg)	58.7 (10.5)	58.8 (6.7)	−0.08 (66)	0.937	60.8 (10.7)	63.0 (9.0)	−1.41 (60)	0.165	1.132	1.01	0.317	0.01	0.907	1.01	0.317
HR (/min)	68.3 (12.5)	66.0 (12.0)	1.10 (66)	0.254	68.2 (9.2)	65.6 (8.7)	1.74 (60)	0.087	1.132	0.32	0.573	0.03	0.863	0.13	0.719
BT (°/C)	36.5 (0.4)	36.5 (0.3)	−0.31 (65)	0.741	36.6 (0.4)	36.5 (0.3)	0.63 (60)	0.533	1.131	1.58	0.211	0.47	0.497	1.56	0.214
WBC (10^3^/mm^3^)	4.0 (1.0)	4.5 (1.4)	−2.94 (56)	0.004 **	3.96 (1.1)	4.19 (1.2)	−1.51 (39)	0.138	1.94	4.90	0.029 *	0.75	0.387	0.47	0.496
Hb (g/dL)	12.3 (1.6)	11.7 (1.2)	3.42 (56)	0.001 **	11.3 (1.5)	11.4 (1.2)	−0.64 (39)	0.529	1.94	9.86	0.002 **	4.25	0.042 *	2.27	0.135
PLT (10^3^/mm^3^)	219.1 (70.4)	249.7 (66.9)	−2.42 (56)	0.019 *	229.9 (76.8)	234.3 (61.8)	−0.45 (39)	0.657	1.94	0.44	0.510	15.57	<0.001 ***	0.173	0.678
Na (mEq/L)	139.5 (3.1)	141.0 (2.1)	−2.81 (41)	0.008 **	139.7 (2.8)	140.7 (2.2)	−0.16 (31)	0.142	1.71	0.02	0.881	1.17	0.283	0.00	0.959
K (mEq/L)	4.2 (0.4)	4.2 (0.3)	−0.35 (41)	0.727	3.6 (0.7)	4.0 (0.5)	−3.20 (31)	0.003 **	1.71	5.95	0.017 *	2.28	0.136	2.01	0.161
Cl (mEq/L)	104.5 (4.3)	106.8 (5.0)	−2.45 (41)	0.019 *	101.3 (7.1)	104.5 (4.8)	−2.79 (31)	0.009 **	1.71	4.62	0.035 *	0.26	0.614	0.00	0.976
AST (U/L)	48.6 (85.5)	27.3 (10.3)	1.79 (51)	0.079	31.9 (19.7)	28.5 (18.5)	0.578 (20)	0.570	1.70	0.04	0.840	3.61	0.061	0.00	0.965
ALT (U/L)	52.7 (81.3)	33.7 (24.6)	1.71 (51)	0.093	37.4 (39.6)	33.8 (25.8)	0.389 (20)	0.702	1.70	0.03	0.863	2.52	0.117	0.01	0.934
Protein (g/dL)	6.6 (0.7)	6.5 (0.5)	0.77 (47)	0.448	6.2 (0.6)	6.4 (0.4)	−0.98 (20)	0.340	1.66	5.78	0.019 *	0.04	0.841	0.95	0.334
Albumin (g/dL)	4.3 (0.5)	4.2 (0.4)	0.55 (49)	0.585	4.1 (0.4)	4.1 (0.3)	0.00 (22)	1.00	1.70	1.36	0.247	0.03	0.876	0.12	0.734
Cholesterol (mg/dL)	199.7 (59.5)	181.2 (30.9)	2.08 (35)	0.045 *	203.9 (63.6)	181.5 (31.0)	1.61 (15)	0.128	1.49	0.17	0.684	0.48	0.490	0.14	0.706
Glucose (mg/dL)	77.3 (13.2)	74.1 (10.5)	1.51 (39)	0.140	70.9 (12.6)	73.1 (11.1)	−0.69 (15)	0.502	1.53	3.15	v082	1.21	0..276	0.60	0.442

Abbreviations: AN, anorexia nervosa; PN, parenteral nutrition; systolic BP, maximal blood pressure; diastolic BP, minimal blood pressure; HR, heart rate; BT, body temperature; WBC, white blood cells; Hb, hemoglobin; PLT, platelets; Na, sodium; K, potassium; CI, chloride; AST, aspartate aminotransferase; ALT, alanine aminotransferase. The effects of PN on the medical findings at discharge were examined with the repeated-measures analysis of variance (ANOVA) model according to the group (patients with PN, patients without PN) and period (admission and discharge) including initial BMI as a covariate. Paired t-tests were used for the group-specific comparisons of medical findings between the periods. * *p* < 0.05, ** *p* < 0.01, *** *p* < 0.001.

**Table 4:** Weight outcomes of AN patients with and without PN at discharge and follow-up after discharge.

	Discharge					Follow-Up at 1 Month					Follow-Up at 3 Months					Follow-Up at 6 Months				
Variables	PN+ (*n* = 67)	PN− (*n* = 62)	F (df = 127)	*p*	Δη^2^	PN+ (*n* = 56)	PN− (*n* = 51)	F (df=105)	*p*	Δη^2^	PN+ (*n* = 45)	PN− (*n* =35)	F (df =78)	*p*	Δη^2^	PN+ (*n* = 34)	PN− (*n* = 29)	F (df = 61)	*p*	Δη^2^
BMI (kg/m^2^)	14.79 (1.77)	16.41 (1.68)	14.47	<0.001 ***	0.096	15.65 (1.99)	16.81 (1.65)	2.27	0.135	0.021	16.92 (2.54)	17.58 (2.60)	0.12	0.727	0.002	17.14 (3.10)	18.55 (2.79)	1.29	0.282	0.040
Weight gain from admission (kg)	3.14 (2.57)	2.19 (1.84)	3.44	0.066	0.025	5.43 (3.67)	3.56 (3.32)	6.95	0.010 **	0.060	9.25 (5.93)	5.89 (5.94)	6.33	0.014 *	0.074	9.67 (8.50)	7.11 (6.65)	0.75	0.388	0.012
Rate (kg/week)	0.88 (0.90)	0.79 (1.02)	0.00	0.961	0.000	0.52 (0.70)	0.33 (0.72)	2.75	0.100	0.025	0.42 (0.50)	0.18 (0.54)	3.64	0.060	0.045	0.12 (0.33)	0.14 (0.30)	0.83	0.365	0.013

Abbreviations: AN, anorexia nervosa, PN, parenteral nutrition; PN+, supplemental parenteral nutrition added to oral refeeding; PN-, oral refeeding alone; BMI, body mass index; Δη^2^, partial eta squared effect size. For comparisons of the groups, ANOVAs with block design were applied to control for the effect of subtypes as the restriction type was higher in the PN+ group. * *p* < 0.05, ** *p* < 0.01, *** *p* < 0.001.
